# Supplementary material for: Appetite-Suppressing and Satiety-Increasing Bioactive Phytochemicals: A Systematic Review
Source: Nutrients. 2019 Sep 17;11(9):2238. doi: 10.3390/nu11092238 (PMC6769678; doi:10.3390/nu11092238)
Supplement: Supplementary file 1 [file nutrients-11-02238-s001.pdf]

Supplementary Materials

Table S1: Search strategy for Ovid MEDLINE®.

	Searches	Results
1	exp Plants/ or exp plant extracts/ or Dietary Supplements/ or Plants, Medicinal/ or exp coffee/ or exp teas, herbal/ or exp Teas, Medicinal/ or exp tea/ or exp Caffeine/ or exp Menthol/ or exp Phenol/ or exp Polyphenols/ or exp Curcumin/ or (herbal or 'phyto nutrient*' or phytonutrient* or 'phyto chemical*' or phytochemical* or 'phyto constituent*' or phytoconstituent* or nutraceutical or 'bioactive ingredient*' or 'bioactive compound*').ti,ab. or (dietary adj1 (constituent* or supplement*)).ti,ab. or (tea* or coffee* or caffein* or coffein* or mate).ti,ab. or ('caralluma fimbriata' or 'hoodia gordonii' or 'garcinia cambogia' or succulent* or 'citrus aurantium' or 'catha edulis' or khat or 'coleus forskohlii' or forskolin or saffron* or umeboshi or flaxseed* or 'linum usitatissimum' or ginger or 'zingiber* officinale' or almond* or aloe or pineapple* or psyllium or 'plantago ovata' or 'capsicum annum' or pepper* or 'harpagophytum procumbens' or ginseng* or 'camellia sinensis' or 'coix lacryma-jobi' or 'gymnema sylvestre' or 'cyamopsis tetragonolobus' or 'punica grantum' or 'amorphophallus konjac' or 'benincasa hispida' or 'mitragyna speciosa' or 'cissus quadrangularis' or 'ephedra sinica' or 'robinia pseudoacacia' or 'evodiae fructus' or 'eucommia' or 'ilex paraguariensis' or plant* or extract* or mint or menthol* or phenol* or polyphenol* or stilbene* or curcumin* or coumarin or flav* or isoflavon* or lignan* or terpen* or carotenoid* or capsaicin* or piperine* or theophylline* or theobromine* or 'hydroxycitric acid*' or organosulfur* or phytosterol* or alkaloid* or chalcone* or sesquiterpene* or xanthine* or alkylamide* or anthocyanin*).ti,ab.	2325576
2	Appetite/ or exp Hunger/ or exp Satiation/ or Appetite Regulation/ or ((appetite or hunger) adj3 (control* or suppress* or reduc* or regulat*)).ti,ab. or satiety.ti,ab. or fullness.ti,ab.	30245
3	Weight Loss/ or exp Overweight/ or exp Energy Intake/ or 'weight loss'.ti,ab. or 'weight management'.ti,ab. or obesity.ti,ab. or 'energy balance'.ti,ab. or ((food or energy or calor*) adj3 intake).ti,ab.	428453
4	1 and 2 and 3	1364
5	limit 4 to (english or german)	1340
6	5 not (animals not humans).sh.	1062

Supplementary Materials

Table S2: Baseline characteristics [1].

Study	Year	Type of study	Plant extract(s) of [2]	Primary outcome scale	Participant, n	Age, years (SD)	Female, %	BMI, kg/m2 (SD)	WHR	Follow-up, days [3]	Follow-up, minutes [3]
Auvichayapat et al. [48]	2008	RCT	*Camellia sinensis* (green tea)	VAS	60	48.7 (5.2)	70	27.7 (3.4)	0.86	84	n.a.
Boix-Castejon et al. [71]	2018	RCT	*Hibiscus sabdariffa* + *Aloysia citriodora*	VAS	54	51.0 (n.r.)	100	29.7 (3.8)	0.90	60	n.a.
Diepvens et al. [49]	2005	RCT	*Camellia sinensis* (green tea)	VAS	46	41.7 (9.3)	100	27.7 (1.8)	0.80	87	180
Dostal et al. [50]	2017	RCT	*Camellia sinensis* (green tea)	VAS	64	60.9 (n.r.)	100	28.3 (n.r.)	0.86	365	240
Gonzalez et al. [68]	2018	RCT	*Crocus sativus* + *Citrus paradisi*	VAS	20	25.5 (3.8)	n.r.	29.9 (5.1)	0.89	28	n.a.
Gout et al. [67]	2010	RCT	*Crocus sativus*	% of participants	61	36.1 (0.7)	100	26.8 (0.2)	0.85	56	n.a.
Kazemipoor et al. [15]	2016	RCT	*Carum carvi*	VAS	70	36.1 (0.7)	100	n.r.	0.90	90	n.a.
Kudiganti et al. [74]	2016	RCT	*Sphaeranthus indicus* + *Garcinia mangostana*	VAS	60	38.0 (1.7)	63.2	28.3 (0.2)	0.95	112	n.a.
Kuriyan et al. [66]	2007	RCT	*Caralluma adscendens var. fimbriata*	VAS	62	38.8 (7.0)	78	30.2 (4.8)	0.90	60	n.a.
Lejeune et al. [59]	2003	RCT	*Capsicum annuum*	VAS	120	n.r.	74.7	27.0 (8.0)	0.85	91	n.a.
Mangine et al. [55]	2012	RCT	*Camellia sinensis* (green tea)	HSS	50	33.5 (13.2)	71.9	33.3 (6.5)	n.r.	56	n.a.
Rondanelli et al. [64]	2013	RCT	*Camellia sinensis* (green tea) + *Capsicum annuum* + *Piper nigrum* + *Fucus vesiculosus* + *Allium sativa*	Haber	37	43.7 (9.2)	73	30.3 (3.0)	0.90	56	n.a.
Rondanelli et al. [72]	2011	RCT	*Phaseolus vulgaris* + *Cynara scolymus*	Haber	40	49.8 (8.0)	61.5	31.3 (2.5)	n.r.	60	n.a.
Rondanelli et al. [56]	2009	RCT	*Camellia sinensis* (green tea)	Haber	138	39.3 (10.4)	91.4	n.r.	n.r.	56	n.a.
Roshan et al. [63]	2018	RCT	*Coffea* sp.	SNAQ	50	52.3 (9.3)	76.7	n.r.	n.r.	64	n.a.
Urbina et al. [58]	2017	RCT	*Capsicum annuum*	CNAQ	111	30.0 (1.0)	59.7	27.5 (0.6)	0.86	84	n.a.
Westerterp-Plantenga et al. [54]	2005	RCT	*Camellia sinensis* (green tea)	VAS	76	n.r.	n.r.	27.5 (2.7)	n.r.	91	n.a.
Alkhatib et al. [65]	2015	Co-RCT	*Camellia sinensis* (green tea) + *Ilex paraguariensis* (Yerba Maté) + *Paullinia cupana* + *Coffea* sp. + *Serenoa repens* + *Polygonum multiflorum* +	Hunger scale	12	24 (3.8)	41.7	22.5 (3.9)	n.r.	n.a.	120

Supplementary Materials

Study	Year	Type of study	Plant extract(s) of [2]	Primary outcome scale	Participant, n	Age, years (SD)	Female, %	BMI, kg/m2 (SD)	WHR	Follow-up, days [3]	Follow-up, minutes [3]
			Eleutherococcus senticosus + *Capsicum annuum* + *Pausinystalia yohimbe*								
Alkhatib et al. [28]	2017	Co-RCT	*Ilex paraguariensis* (Yerba Maté)	VAS	21	30.8 (7.3)	100	22.0 (1.1)	n.r.	n.a.	180
Fernandes et al. [51]	2018	Co-RCT	*Camellia sinensis* (green tea)	VAS	23	24.4 (0.6)	95.7	21.1 (0.4)	0.74	n.a.	150
Greenberg et al. [75]	2016	Co-RCT	*Theobroma cacao*	VAS	30	22.7 (3.9)	0	23.3 (2.4)	0.81	n.a.	150
Greenberg et al. [60]	2012	Co-RCT	*Coffea* sp.	VAS	11	23.5 (5.7)	0	23.6 (4.2)	n.r.	n.a.	180
Hao et al. [73]	2017	Co-RCT	*Salacia chinensis*	VAS	54	32.0 (12.0)	58.3	28.8 (3.6)	n.r.	n.a.	180
Hochkogler et al. [57]	2014	Co-RCT	*Capsicum annuum*	VAS	15	25 (20-39)	0	27.5 (1.5)	n.r.	n.a.	120
Hochkogler et al. [69]	2017	Co-RCT	*Eriodictyon californicum*	VAS	24	25.9 (4.5)	64.7	21.6 (2.3)	n.r.	n.a.	120
Janssens et al. [21]	2014	Co-RCT	*Capsicum frutescens* + *Capsicum annuum*	VAS	19	29.7 (10.8)	46.7	23.3 (2.9)	0.75	n.a.	2160
Josic et al. [52]	2010	Co-RCT	*Camellia sinensis* (green tea)	VAS, Haber	15	27.0 (3.0)	50.0	22.3 (3.4)	n.r.	n.a.	120
Mennella et al. [70]	2016	Co-RCT	*Gentiana lutea*	VAS	20	25.3 (3.1)	45.0	22.1 (2.3)	n.r.	n.a.	180
Panek-Shirley et al. [61]	2018	Co-RCT	*Coffea* sp.	Likert Scale	53	25.2 (1.5)	58.0	24.5 (2.7)	0.8 (0.1)	n.a.	60
Reinbach et al. [53]	2009	Co-RCT	I1: *Capsicum annuum* (cayenne)<br>I2: *Camellia sinensis* (green tea)<br>I3: *Capsicum annuum* (CH-19 sweet pepper)<br>I4: *Capsicum annuum* (cayenne) + *Camellia sinensis* (green tea)	VAS	27	26.9 (6.3)	62.9	22.2 (2.7)	n.r.	21	n.a.
Schubert et al. [62]	2014	Co-RCT	*Coffea* sp.	VAS	18	26.3 (6.3)	75.0	22.7 (2.2)	n.r.	n.a.	270
Shin et al. [76]	2015	Co-RCT	*Vitis vinifera*	VAS	20	26.4 (1.7)	0	23.1 (0.7)	n.r.	n.a.	330

[1] CNAQ, Council on Nutrition Appetite Questionnaire; Co-RCT, crossover randomized controlled trial; HSS, Hunger and Satiety Scale; LMS, labelled magnitude satiety scale; n.a., not applicable; n.r., not reported, RCT, randomized controlled trial; SNAQ, Simplified Nutritional Appetite Questionnaire; VAS, visual analogue scale; WHR, waist/hip-ratio. [2] For accepted scientific name and exact composition see Table S4. [3] Three different ways of reporting: measurements over several days: there is a value for follow-up days, but no value for follow-up minutes; measurements over several minutes on just one day: there is a value for follow-up minutes, but no value for follow-up days; measurements over several days and over several minutes on each of these days: there are values for both follow-up days and follow-up minutes.

Supplementary Materials

Table S3: Study characteristics [1.]

Study	Year	Type of study	Study location	Number of sites	Inclusion criteria	Exclusion criteria	Outwashing, days
Auvichayapat et al. [48]	2008	RCT	Faculty of Medicine of Khon Kaen University, Thailand	1	1) Males, aged 40 to 60 years<br>2) Females, postmenopausal > 1 year<br>3) BMI > 25 kg/m^2	1) Metabolic disease, such as diabetes mellitus, hyper- or hypo thyroidism, and Cushing syndrome<br>2) Systemic disease, such as heart-, renal-, or liver disease<br>3) Use of prescribed medications, such as antipsychotics, antidepressants, antiobesity medications, or hormonal therapy<br>4) Regular exercise or an average total energy expenditure > 8373.6 kJ/day<br>5) History of tea or caffeine hypersensitivity	n.a.
Boix-Castejon et al. [71]	2018	RCT	Alicante, Spain	n.r.	1) Females<br>2) BMI 25 to 34.9 kg/m^2	1) Any obesity-related pathology<br>2) Use of prescribed medication for hypercholesterolemia or hypertension<br>3) Consumption of antioxidant supplements/drugs<br>4) Frequent alcohol consumption<br>5) Pregnant/lactating	n.a.
Diepvens et al. [49]	2005	RCT	n.r.	n.r.	1) Age 19 to 57 years<br>2) BMI 25 to 31 kg/m^2<br>3) Moderate caffeine-users (200 - 400 mg caffeine/d)<br>4) Good health<br>5) Non-smokers<br>6) Normotensive<br>7) No use of medication<br>8) At most moderate alcohol users	n.r.	n.a.
Dostal et al. [50]	2017	RCT	University of Minnesota's Delaware Clinical Research Unit, USA	1	1) Healthy<br>2) Nonsmoking<br>3) Post-menopausal women<br>4) Age 50 to 70 years<br>5) Classified as having 'heterogeneously dense' or 'extremely dense' breast tissue after a routine screening mammogram<br>6) BMI > 25kg/m^2	n.r.	n.a.
Gonzalez et al. [68]	2018	RCT	Hofstra University, NY, USA	1	1) Age 18 to 59 years<br>2) BMI > 25 kg/m^2<br>3) Consuming at least two large meals per day	1) Use of other medication (e.g., ADHD medication, antidepressants, antibiotics, etc.)	n.a.

Supplementary Materials

Study	Year	Type of study	Study location	Number of sites	Inclusion criteria	Exclusion criteria	Outwashing, days
					4) No particular diet plan (e.g., low carbohydrate, ketogenic, vegan, gluten free, intermittent fasting, etc.)	2) Use of nutritional supplements (including multivitamins)	
Gout et al. [67]	2010	RCT	GSR Investigation Center, Toulouse, France	1	1) Healthy women<br>2) Age 25 to 45 years<br>3) BMI 25 kg/m^2 to 28 kg/m^2	1) Cancer<br>2) Diabetes<br>3) Pathologic eating disorders (anorexia and bulimia nervosa)<br>4) Anxiety or depression<br>5) Abnormal liver or renal function<br>6) Use of psychotropic drugs or appetite suppressants<br>7) Gastric surgery<br>8) Use of any food supplement that might interfere with the study results	n.a.
Kazemipoor et al. [15]	2016	RCT	n.r.	n.r.	1) Habitually performing aerobics during the whole period of the intervention	n.r.	n.a.
Kudiganti et al. [74]	2016	RCT	Srinivasa Clinic & Diabetic Care Centre, Southern India	1	1) Healthy overweight adult men and women<br>2) Age 21 to 50 years<br>3) Willing to adhere to a vegetarian or non-vegetarian diet<br>4) Willing to walk for 5 days per week<br>5) BMI 27 to 32 kg/m^2	1) Intractable obesity<br>2) History of chronic diseases, or personal behaviors that would confound the interpretation of results arising from this study	n.a.
Kuriyan et al. [66]	2007	RCT	Nutrition Clinic of St. John's Medical College Hospital, Bangalore, India	1	1) Age 25 to 60 years<br>2) BMI > 25 kg/m^2	1) Presence of any chronic disease<br>2) Use of any medication for weight loss	n.a.
Lejeune et al. [59]	2003	RCT	n.r.	n.r.	1) Age 18 to 60 years<br>2) BMI 25 to 35 kg/m^2<br>3) Good health<br>4) Nonsmoking<br>5) No use of medication<br>6) At most moderate alcohol user	n.r.	n.a.
Mangine et al. [55]	2012	RCT	n.r.	n.r.	1) Age 18 to 59 years<br>2) BMI 25 to 40 kg/m^2<br>3) Daily energy intake to be at, or above their calculated dietary fuel requirement	n.r.	n.a.
Rondanelli et al. [64]	2013	1) RCT	2) Endocrinology and Clinical Nutrition Unit of Azienda di Servizi alla Persona di Pavia, University of Pavia, Italy	2	1) Healthy men and women<br>2) Age 25 to 45 years<br>3) BMI 25 to 35 kg/m^2	1) Any hepatic or renal disease<br>2) Diabetes, unstable cardiovascular disease or uncontrolled hypertension<br>3) Eating disorder (diagnosed bulimia)<br>4) Active cancer	n.a.

Supplementary Materials

Study	Year	Type of study	Study location	Number of sites	Inclusion criteria	Exclusion criteria	Outwashing, days
			3) Dietetic and Metabolic Unit, "Villa delle Querce" Clinical Rehabilitation Institute, Rome, Italy			5) Undergone surgery for weight loss<br>6) Major depressive disorder<br>7) Use of medications for weight loss<br>8) Pregnant or lactating<br>9) Entered menopause	
Rondanelli et al. [72]	2011	RCT	Outpatient Dietetic and Metabolic Unit, Azienda di Servizi alla Persona, University of Pavia, Italy	1	1) Age 18 to 50 years<br>2) BMI 25 to 35 kg/m^2<br>3) Females were required to be premenopausal, not currently pregnant and normally menstruating	1) Critical alterations in lipid and carbohydrate metabolism (cholesterol > 6.21 mmol/L, glucose > 7.8 mmol/L)<br>2) Acute or disabling conditions<br>3) Endocrinological-, neoplastic- or autoimmune diseases<br>4) History, signs, or symptoms of heart disease<br>5) Major depressive disorder<br>6) History or current diagnosis of bulimia, panic disorder, obsessive-compulsive disorder, post-traumatic stress disorder, bipolar I or II disorder or schizophrenia<br>7) Use of psychoactive or antiobesity drugs	n.a.
Rondanelli et al. [56]	2009	RCT	Outpatient Unit for the Treatment of Obesity, Fondazione IRCCS Policlinico San Matteo, Presidio di Belgioioso, University of Pavia, Italy	1	1) Age 18 to 50 years<br>2) Not pregnant and normally menstruating<br>3) BMI 25 to 35 kg/m^2<br>4) No significant alterations in lipid and carbohydrate metabolism (glucose 6.11 mmol/l, total cholesterol 6.20 mmol/l, TAG 2.28 mmol/l)<br>5) Any acute or disabling conditions<br>6) Any endocrinological-, neoplastic- and autoimmune disease<br>7) No history, signs or symptoms of heart disease (mild hypertension with systolic pressure 140 – 150 mmHg and diastolic pressure 80 – 95 mmHg was allowed	1) Major depressive disorder<br>2) History or current diagnosis of bulimia, panic disorder, obsessive compulsive disorder, post-traumatic stress disorder, bipolar I or II disorder, or schizophrenia<br>3) Use of psychoactive drugs, including anti-obesity agents	n.a.
Roshan et al. [63]	2018	RCT	Imam Hossein Hospital diabetes clinic, Tehran, Iran	1	1) Age 18 to 70 years<br>2) BMI > 25 kg/m^2<br>3) Metabolic syndrome (according to the new International Diabetes Federation definition: central obesity (waist circumference > 102 cm in men or > 88 cm in women) in conjunction with two of the following risk factors: fasting blood glucose	1) Insulin administration for controlling blood glucose<br>2) Hypo- or hyperthyroidism<br>3) Renal failure<br>4) Routine coffee consumption<br>5) Pregnancy or breast-feeding	n.a.

Supplementary Materials

Study	Year	Type of study	Study location	Number of sites	Inclusion criteria	Exclusion criteria	Outwashing, days
					> 100 mg/dl (> 5.55 mmol/l), TAG > 150 mg/dl (> 1.6 mmol/l), HDL-cholesterol < 50 mg/dl (< 1.29 mmol/l) in women or < 40 mg/dl (< 1.03 mmol/l) in men, systolic blood pressure > 130 mmHg and diastolic blood pressure > 85 mmHg)	6) Use of corticosteroids, hormone replacement therapy as oestrogen or progesterone or weight loss supplements<br>7) Following unusual weight loss plans<br>8) Cancer<br>9) Experiencing cerebrovascular accident and other cognitive problems or chronic diseases that impaired their compliance<br>10) Alteration of the medication controlling blood glucose, blood pressure or lipid profile<br>11) Not consumed over 10 % of the supplements	
Urbina et al. [58]	2017	RCT	n.r.	n.r.	1) Age 18 to 56 years<br>2) Apparently healthy and free from disease<br>3) No use of any ergogenic supplements in the last 6 months<br>4) Able to do everything required in the study<br>5) Agree to not do strenuous activity 24 to 48 hours before appointment<br>6) Agree to not smoke or use caffeine and tobacco for 12 hours before appointment<br>7) Agree to not eat or drink anything for 12 hours before appointment<br>8) Agree to not drink alcohol 24 hours before appointment<br>9) BMI 24.5 to 29.5 kg/m^2	n.r.	n.a.
Westerterp-Plantenga et al. [54]	2005	RCT	n.r.	n.r.	1) Age 18 to 60 years<br>2) BMI 25 to 35 kg/m^2<br>3) Good health<br>4) Nonsmoker<br>5) No use of medication<br>6) At most a moderate alcohol user	n.r.	n.a.
Alkhatib et al. [65]	2015	Co-RCT	n.r.	n.r.	1) Free from illness and any type of orthopedic limitation or injury	1) History of any cardiovascular- or respiratory disease, hypertension, liver- or kidney disease, musculoskeletal- or neuromuscular- or neurological disease, autoimmune disease, cancer, peptic ulcers or anemia<br>2) Use of medications (except contraceptive pills), including those for	3

Supplementary Materials

Study	Year	Type of study	Study location	Number of sites	Inclusion criteria	Exclusion criteria	Outwashing, days
						heart, pulmonary, thyroid, anti-hyperlipidemic, hypoglycemic, anti-hypertensive, endocrinologic, psychotropic, neuromuscular, neurological, or androgenic conditions<br>3) Family history of heart problems, high blood pressure, and/or stroke<br>4) Pregnant or breastfeeding<br>1) Consuming any ergogenic aid or above habitual caffeine consumption rate (200 mg/d) for at least 6 weeks prior to the study, based on all types of caffeinated beverages (coffee, energy drinks, soft drinks, caffeine supplements or medications)	
Alkhatib et al. [28]	2017	Co-RCT	n.r.	n.r.	1) Female<br>2) Age 18 to 40 years<br>3) Habitually complete 150 minutes of moderate physical activity per week<br>4) BMI < 30kg/m^2	1) History of any cardiovascular- or respiratory disease, hypertension, liver- or kidney disease, musculoskeletal- or neuromuscular- or neurological disease, autoimmune disease, cancer, peptic ulcers or anemia<br>2) Use of medications, including those for heart, pulmonary, thyroid, anti-hyperlipidemic, hypoglycemic, anti-hypertensive, endocrinologic, psychotropic, neuromuscular, neurological, or androgenic conditions<br>3) Family history of heart problems, high blood pressure, and/or stroke<br>4) Pregnant or breastfeeding<br>5) Consuming any ergogenic aid or above habitual caffeine consumption rate (200 mg/d) for at least 6 weeks prior to the study, based on all types of caffeinated beverages (coffee, energy drinks, soft drinks, caffeine supplements, or medications)	3
Fernandes et al. [51]	2018	Co-RCT	n.r.	n.r.	1) Healthy<br>2) Female<br>3) Stable weight in the last 12 months (weight gain or loss less than 5 %)	1) Asian descent<br>2) BMI < 18.5 or > 24.9 kg/m^2<br>3) Autoimmune diseases<br>4) Use of immunosuppressive drugs	7

Supplementary Materials

Study	Year	Type of study	Study location	Number of sites	Inclusion criteria	Exclusion criteria	Outwashing, days
						5) Diabetes, thyroid dysfunction, chronic kidney disease or liver disease<br>6) Undergone bariatric surgery<br>7) Chronic alcoholics or smokers<br>8) Use of hormonal medications (e.g., contraceptives) or appetite/body weight-management drugs (e.g., appetite suppressants) in the last 12 months<br>9) Use of pump inhibitor medications<br>10) Participated in any food restriction program<br>11) Use of nutritional supplements during the past 12 months<br>12) Lactose or fructose intolerant	
Greenberg et al. [75]	2016	Co-RCT	Brooklyn College, City University of New York, USA	1	1) Nonsmokers<br>2) BMI 18.5 to 30 kg/m^2<br>3) Stable weight (a gain or loss of < 5 % in the past 6 month)<br>4) Moderate alcohol users (< 2 drinks/d)<br>5) Mentally and physically healthy<br>6) Willing to consume pizza and cocoa beverages	1) Being regular, frequent drinkers of coffee, tea, or sodas that contained caffeine (> 1 serving/d)<br>2) Participating in regular, frequent vigorous physical activity<br>3) Use of medication that could affect appetite<br>4) Being allergic to chocolate, cocoa, or pizza<br>5) Attempting to gain or lose weight<br>6) Interested in registering for courses taught by the principal investigator<br>7) Women (to avoid possible effects of menstrual hormones on appetite)	> 7
Greenberg et al. [60]	2012	Co-RCT	n.r.	n.r.	1) Healthy<br>2) Male<br>3) Nonsmoker<br>4) No use of medications that could influence body weight or interfere with glucose metabolism	n.r.	7 to 14
Hao et al. [73]	2017	Co-RCT	Department of Nutritional Sciences, Rutgers University, New Jersey, USA	1	1) Healthy<br>2) BMI 25 to 35 kg/m^2	1) Eating disorder<br>2) Gastrointestinal illness or bariatric surgery<br>3) Hyperparathyroidism or untreated thyroid disease<br>4) Diabetes<br>5) Blood pressure > 140/90 mmHg	30

Supplementary Materials

Study	Year	Type of study	Study location	Number of sites	Inclusion criteria	Exclusion criteria	Outwashing, days
						6) Significant immune, hepatic, or renal disease<br>7) Significant cardiac disease<br>8) Active malignancy or cancer therapy within the past year<br>9) Use of obesity medications or dietary supplements or any weight regimen	
Hochkogler et al. [57]	2014	Co-RCT	University of Vienna, Vienna, Austria	1	1) Age 20 to 40 years<br>2) BMI 25 to 32 kg/m^2<br>3) Nonsmoking<br>4) No abnormal eating behavior<br>5) No alcohol abuse<br>6) No medication<br>7) Metabolically healthy<br>8) Sensorially untrained<br>9) Fasting blood glucose < 120 mg/dL	1) Women (due to their menstrual cycle as estrogen has been shown to interfere with serotonin concentrations)	> 7
Hochkogler et al. [69]	2017	Co-RCT	University of Vienna, Vienna, Austria	1	1) Age 20 to 45 years<br>2) BMI 18.5 to 25 kg/m^2<br>3) Nonsmoking<br>4) No alcohol- or drug abuse<br>5) Metabolically healthy<br>6) Sensorially untrained	n.r.	7
Janssens et al. [21]	2014	Co-RCT	Metabolic unit of Maastricht University, department of Human Biology, Netherlands	1	7) Age 18 to 50 years<br>8) BMI 20 to 30 kg/m^2<br>9) Good health<br>10) Nonsmoking<br>11) No use of dietary supplement and medication except for oral contraceptives<br>12) Not more than moderate amount of alcohol (less than 10 g alcohol per drink, less then 10 drinks per week) or caffeine-containing beverages (less than 2 cups per day)<br>13) Weight stable (weight change < 3 kg during the last 6 months) and dietary unrestrained<br>14) Lightly or moderately active (1 to 5 hours moderate exercise per week)<br>15) Used to spicy foods on a regular basis (1 to 2 days per week, in a low dosage with one meal/day)	1) Pregnant or lactating women<br>2) Allergies for the food items used in the study	men: > 7<br>women: > 28
Josic et al. [52]	2010	Co-RCT	Clinical research department, Skåne	1	1) Healthy<br>2) Normal weight	n.r.	> 7

Supplementary Materials

Study	Year	Type of study	Study location	Number of sites	Inclusion criteria	Exclusion criteria	Outwashing, days
			University Hospital, Malmö, Sweden				
Mennella et al. [70]	2016	Co-RCT	Department of Agricultural Sciences of Naples, USA	1	n.r.	1) BMI ≥ 25 kg/m^2<br>2) Any chronic illnesses such as diabetes or hypertension<br>3) Smokers<br>4) Use of prescription medication<br>5) Under a controlled dietary regimen<br>6) Lost weight over the previous 3 months<br>7) Pregnant or lactating	7
Panek-Shirley et al. [61]	2018	Co-RCT	University at Buffalo, Department of Exercise and Nutrition Sciences, Nutrition and Health Research Laboratory, USA	1	1) Age 18 to 50 years	1) No previous experience or a previous adverse event with caffeine<br>2) Use of any medication<br>3) Medical condition contraindicating caffeine or stimulant consumption<br>4) Medical condition affecting appetite or eating<br>5) Use of tobacco products within the past 6 months	7
Reinbach et al. [53]	2009	Co-RCT	Maastricht University Hospital, Netherlands	1	n.r.	n.r.	7
Schubert et al. [62]	2014	Co-RCT	Helsinki, Sweden	n.r.	1) Non-smoking<br>2) Pre-menopausal women<br>3) BMI < 30 kg/m^2<br>4) Age 18 to 45 years<br>5) No use of any medicine known to influence lipid, carbohydrate, or caffeine metabolism<br>6) Not dieting and no extreme dietary behaviours (Three Factor Eating Questionnaire)<br>7) Weight stable in the previous 3 months (± 5 % by self-report)<br>8) No history of any cardiovascular- or metabolic diseases<br>9) No food allergies or intolerances<br>10) No history of gastrointestinal disorders	1) Atypical or abnormal eating patterns which could have potentially confounded the study outcomes	3 to 4
Shin et al. [76]	2015	Co-RCT	Human Nutrition Unit, University of Auckland, New Zealand	1	1) Healthy men<br>2) BMI 18 to 28kg/m^2<br>3) Age 18 to 60 years<br>4) Nonsmokers	1) Active diet program or loss/gain of > 5 kg within the last 6 months<br>2) Hypersensitivities or allergies to any foods or ingredients included in the study	3

Supplementary Materials

Study	Year	Type of study	Study location	Number of sites	Inclusion criteria	Exclusion criteria	Outwashing, days
					5) No history of cardiovascular disease, diabetes, or any other significant metabolic, endocrine or gastrointestinal disease<br>6) No use of any medications that may have had any effect on appetite or weight regulation throughout the trial period	3) Dislike and/or unwillingness to consume items listed as study foods (breakfast and lunch meals)<br>4) Unwilling/unable to comply with study protocol<br>5) Current participation in another clinical intervention trial	

[1] Co-RCT, crossover randomized controlled trial; n.a., not applicable; n.r., not reported; oGTT, oral **glucose tolerance test; RCT, r**andomized controlled trial; **TFEQ,** Three Factor Eating Questionnaire.

Supplementary Materials

Table S4: Composition of the bioactive phytochemicals in the included studies [1].

Author	Year	Type of study	Commercial product name	Plant of origin	Bioactive phytochemical(s)	Additional compounds/ excipients	Placebo	Form
Auvichayapat et al. [48]	2008	RCT	n.r.	*Camellia sinensis* L. Kuntze (green tea)	250 mg green tea leaf extracts, thereof: 4.09 mg catechin, 33.58 mg EGCG, 9.28 mg ECG, 28.86 mg caffeine, 0.24 mg gallic acid	n.r.	Cellulose	Capsule
Boix-Castejon et al. [71]	2018	RCT	MetabolAid®	*Hibiscus sabdariffa* L. (65 %) + *Aloysia citriodora* Palau (syn. *Lippia citriodora) (35 %)*	500 mg polyphenolic extracts, thereof: 3.5 % anthocyanins, 15 % verbascoside	n.r.	Crystalline microcellulose	Capsule
Diepvens et al. [49]	2005	RCT	Sunphenon® 100S	*Camellia sinensis* L. Kuntze (green tea)	310 mg green tea extracts, thereof: 134.1 mg total catechins, 14.0 mg EC, 26.7 mg EGC, 23.6 mg ECG, 66.2 mg EGCG, 26.3 mg caffeine	74.9 mg maltodextrin, 71.7 mg microcrystalline cellulose, 1.5 mg silicium dioxide, 1.5 mg magnesium stearate	310 mg maltodextrin	Capsule
Dostal et al. [50]	2017	RCT	n.r.	*Camellia sinensis* L. Kuntze (green tea)	Decaffeinated green tea extracts, thereof: 328.75 mg catechins (210.75 mg as EGCG)	n.r.	816 mg maltodextrin, 808 mg cellulose, 8 mg magnesium stearate	Capsule
Gonzalez et al. [68]	2018	RCT	CraveFix 96	*Crocus sativus* L.<br><br>*Citrus paradisi* Macfad,	89 mg unsp. saffron stigma extracts<br>50 mg naringin	1000 IU Vitamin D3	Rice flour	Capsule
Gout et al. [67]	2010	RCT	Satiereal®	*Crocus sativus* L.	88.25 mg unsp. saffron stigma extracts	Maltodextrin, magnesium stearate, hydrated silica	88.25 mg microcrystalline cellulose, maltodextrin magnesium stearate, hydrated silica	Capsule
Kazemipoor et al. [15]	2016	RCT	n.r.	*Carum carvi* L.	30 ml unsp. caraway aqueous extracts	n.r.	30 ml caraway essence (1 % g/L)	Beverage (30 ml water)
Kudiganti et al. [74]	2016	RCT	Meratrim®	*Sphaeranthus indicus* L.<br>*Garcinia mangostana* L.	300 mg unsp. extracts<br>100 mg unsp. extracts	Microcrystalline cellulose, magnesium stearate	Microcrystalline cellulose, magnesium stearate	Capsule
Kuriyan et al. [66]	2007	RCT	n.r.	*Caralluma adscendens var.*	500 mg unsp. extracts	n.r.	500 mg maltodextrin	Capsule

Supplementary Materials

Author	Year	Type of study	Commercial product name	Plant of origin	Bioactive phytochemical(s)	Additional compounds/ excipients	Placebo	Form
				fimbriata (Wall.) Gravely & Mayur.				
Lejeune et al. [59]	2003	RCT	n.r.	*Capsicum annuum* L.	22.5 mg capsaicin	202.5 mg vegetable oil	225 mg vegetable oil	Capsule
Mangine et al. [55]	2012	RCT	PhosphoLean™	*Camellia sinensis* L. Kuntze (green tea)	35 mg EGCG	40 mg NOPE, 25 mg mixed phospholipids	100 mg rice flour	Capsule
Rondanelli et al. [64]	2013	RCT	n.r.	*Camellia sinensis* L. Kuntze (green tea)<br>*Capsicum annuum* L.<br>*Piper nigrum* L.<br><br>*Fucus vesiculosus* L. [2]<br>*Allium sativa* L.	150 mg decaffeinated unsp. extracts<br><br>7.5 mg oleoresin<br><br>3 mg unsp. extracts<br>56.5 mg unsp. extracts<br>2.5 mg unsp. extracts	150 mg L-Carnitine,<br>2.5 mg mint essential oil	n.r.	Capsule
Rondanelli et al. [72]	2011	RCT	BONVIT®	*Phaseolus vulgaris* L. *Cynara scolymus* L.	100 mg stand. extracts<br>200 mg stand. extracts	n.r.	n.r.	Tablets
Rondanelli et al. [56]	2009	RCT	PhosphoLEAN™	*Camellia sinensis* L. Kuntze (green tea)	50 mg EGCG	85 mg NOPE	n.r.	Capsule
Roshan et al. [63]	2018	RCT	n.r.	*Coffea* sp.	400 mg decaffeinated green coffee bean extracts, thereof: 186 mg chlorogenic acids	n.r.	Starch	Capsule
Urbina et al. [58]	2017	RCT	Capsimax®	*Capsicum annuum* L.	I1: 2 mg capsaicinoids<br>I2: 4 mg capsaicinoids	n.r.	Corn starch	Pill
Westerterp-Plantenga et al. [54]	2005	RCT	n.r.	*Camellia sinensis* L. Kuntze (green tea)	45 mg EGCG, 25 mg caffeine	380 mg vegetable oil	450 ml vegetable oil	Capsule
Alkhatib et al. [65]	2015	Co-RCT	Shred-Matrix®	*Camellia sinensis* L. Kuntze (green tea)<br>*Ilex paraguariensis* A. St.-Hil. (Yerba Maté) *Paullinia cupana* Kunth<br><br>*Coffea* sp.<br><br>*Serenoa repens* (W. Bartram) Small	70 mg unsp. green tea leaf extracts<br><br>Yerba Maté<br><br>100 mg unsp. guarana seed extracts<br>50 mg anhydrous caffeine<br>Saw palmetto	n.r.	Maltodextrin, hemp protein powder	Capsule

Supplementary Materials

Author	Year	Type of study	Commercial product name	Plant of origin	Bioactive phytochemical(s)	Additional compounds/ excipients	Placebo	Form
				Polygonum multiflorum Thunb. *Eleutherococcus senticosus* (Rupr. & Maxim.) Maxim. *Capsicum annuum* L. *Pausinystalia yohimbe* Pierre ex Beille [2]	Fo-Ti<br>Eleuthero root<br>Cayenne pepper<br>Yohimbine HCL			
Alkhatib et al. [28]	2017	Co-RCT	n.r.	*Ilex paraguariensis* A. St.-Hil. (Yerba Maté)	500 mg unsp. Yerba Maté green leaves extracts	n.r.	Empty capsule	Capsule
Fernandes et al. [51]	2018	Co-RCT	Teavigo®	*Camellia sinensis* L. Kuntze (green tea)	376 mg EGCG	n.r.	400 mg corn starch	Capsule (with 295 ml liquid test meal)
Greenberg et al. [60]	2012	Co-RCT	n.r.	*Coffea* sp.	Caffeine:<br>I1: 6 mg per kg body weight<br>I2: 6 mg per kg body weight<br>I3: 0 mg per kg body weight	n.r.	Water	Beverage (I1: Water, I2: Caffeinated coffee, I3: Decaffeinated coffee)
Greenberg et al. [75]	2016	Co-RCT	n.r.	*Theobroma cacao* L.	I1: Nonalkalized cocoa mixture, thereof per kg body weight:<br>0.6 mg EC, 0.2 mg catechin, 2.9 mg procyanidins<br>I2: per kg body weight: 1.0 mg EC<br>I3: per kg body weight: 6.6 mg procyanidins	I2: See placebo<br>I3: See placebo	Alkalized cocoa mixture (0 mg of the compounds of I1, I2, I3)	Beverage (2.96 ml hot water per kg body weight)
Hao et al. [73]	2017	Co-RCT	n.r.	*Salacia chinensis* L.	I1: 300 mg unsp. extracts with α-glucosidase inhibitors properties<br>I2: 500 mg extracts with α-glucosidase inhibitors properties	n.r.	n.r.	Capsule
Hochkogler et al. [57]	2014	Co-RCT	n.r.	*Capsicum annuum* L.	0.15 mg nonivamide (capsaicin analog)	75 g glucose + 15 μL ethanol	75 g glucose + 15 μL ethanol	Beverage (300 ml water)

Supplementary Materials

Author	Year	Type of study	Commercial product name	Plant of origin	Bioactive phytochemical(s)	Additional compounds/ excipients	Placebo	Form
Hochkogler et al. [69]	2017	Co-RCT	n.r.	*Eriodictyon californicum* (Hook. & Arn.) Decne.	30 mg homoeriodyctiol sodium salt	75 g glucose + 15 µL ethanol	75 g glucose + 15 µL ethanol	Beverage (300 ml water)
Janssens et al. [21]	2014	Co-RCT	n.r.	*Capsicum annuum* L.	2.56 mg capsaicin (1.03 g of red chili pepper)	n.r.	None	Beverage
Josic et al. [52]	2010	Co-RCT	n.r.	*Camellia sinensis* L. Kuntze (green tea)	9 g of green tea leaf extracts, thereof: 25.5 mg EC, 89.7 mg ECG, <3 mg EGC, 32.4 mg EGCG	n.r.	300 ml hot water	Beverage (I: 300 ml hot water)
Mennella et al. [70]	2016	Co-RCT	n.r.	*Gentiana lutea* L.	1.25 g root extracts, thereof: 100 mg secoiridoids	n.r.	100 g pudding	Pudding (100 g)
Panek-Shirley et al. [61]	2018	Co-RCT	n.r.	*Coffea* sp.	Powder anhydrous caffeine: I1: 1 mg per kg body weight I2: 3 mg per kg body weight	Caffeine-free lemon-lime-flavored soda	0.1 mg bitter tastant (powder quinine hydrochloride dehydrate) pwer ml caffeine-free lemon-lime-flavored soda	Beverage (350 ml juice)
Reinbach et al. [53]	2009	Co-RCT	n.r.	I1: *Capsicum annuum* L. I2: *Camellia sinensis* L. Kuntze (green tea) I3: *Capsicum annuum* L. (CH-19 sweet pepper) I4: *Capsicum annuum* L. + *Camellia sinensis* L. Kuntze (green tea)	I1: 510 mg cayenne I2: 598.5 mg catechins, 77 mg caffeine as tea I3: 2.3 mg capsiate I4: I1 + I2	n.r.	"placebo capsule" + 3.5 dl water	I1 + I3 + P: Capsule (with 3.5 dl water) I2 + I4: Beverage (3.5 dl water)
Schubert et al. [62] [3]	2014	Co-RCT	n.r.	*Coffea* sp.	I1: 2 mg per kg body weight caffeine I2: 2 mg per kg body weight caffeine + unsp. extracts in coffee	n.r.	Psyllium (Metamucil®)	Capsule (I1 + P: 225 ml water I2: 225 ml decaffeinated coffee)
Shin et al. [76]	2015	Co-RCT	n.r.	*Vitis vinifera* L.	500 mg grape extracts, thereof: 353 mg polyphenols	n.r.	Magnesium stearate	Capsule

Supplementary Materials

[1] 5-HTP, 5-Hydroxytryptophan; Co-RCT, crossover randomized controlled trial; EC, epicatechin; ECG, epicatechin gallate; EGC, epigallocatechin; EGCG, epigallocatechin gallate; I, intervention group; NOPE, N-oleyl-phosphatidylethanolamine; P, placebo group; unsp., unspecified. [2] Yet unresolved in the plant list. [3] Intervention group with psyllium husk and decaffeinated coffee excluded due to our exclusion criteria (seeds).

Supplementary Materials

Table S5: Nutritional regimens [1].

Author	Year	Type of study	Form	Daily intake	Time point of intake	Diet / exercise
Auvichayapat et al. [48]	2008	RCT	Capsule	3 x 1	After breakfast, lunch, and dinner	Thai diet, 3 meals daily with 8373.6 kJ/day (2001 kcal) for 12 weeks containing 65 % carbohydrates, 15 % protein, and 20 % fat.
Boix-Castejon et al. [71]	2018	RCT	Capsule	1 x 1	20 to 30 minutes before breakfast	Participants did not follow a balanced, varied and complete diet. The dietary patterns were isocaloric diet equal in total energy (9250 kJ / 2200 kcal per day energy intake), energy density, dietary fiber and macronutrient with normal hydration. Participants were instructed to walk for at least 30 minutes per day.
Diepvens et al. [49]	2005	RCT	Capsule	3 x 3	At breakfast, lunch and dinner	During days 1 to 87: background caffeine intake was standardized at 300 mg/d in order to maintain their habitual caffeine intake.<br>During days 1 to 3: standardized energy balance diet at 100 % of energy expenditure.<br>During days 4 to 87: low energy diet in order to lose weight. The diet was a meal-replacement diet plan (SlimFast®; Unilever Bestfoods Nederland), in accordance with 60 % of the predicted energy expenditure (energy: protein 25 %, fat 15 %, carbohydrate 60 %), taken at breakfast and lunch. They received instructions about the type and amount of food that they could ingest at dinner and as snacks.
Dostal et al. [50]	2017	RCT	Capsule	2 x 2	With breakfast and in the evening	The day before test day: Adhere to normal energy intake and refrain from exercise and alcohol.<br>Test day: Arrival after a 10 hours fast, consumption of the capsules with a standardized high-carbohydrate breakfast meal (bagel with cream cheese, orange juice and low-fat, fruit-flavored yogurt or 2% milk, 2784.0 kJ / 665.4 kcal, carbohydrate 67.2 %, protein 14.3 %, fat 18.5 %).
Gonzalez et al. [68]	2018	RCT	Capsule	2 x 1	30 minutes before their two biggest meals of the day	Consumption of at least two large meals per day, no active adherence to any particular diet plan (e.g., low carbohydrate, ketogenic, vegan, gluten free, intermittent fasting, etc.). They were allowed to eat ad libitum while enrolled in the study.
Gout et al. [67]	2010	RCT	Capsule	2 x 1	Before breakfast and dinner	Maintenance of usual nutrition regimen and lifestyle.
Kazemipoor et al. [15]	2016	RCT	Beverage	1 x 1	20 minutes before lunch	Habitually performing aerobics during the whole period of the intervention, without changing the dietary habits and the lifestyle patterns.<br>Test day: standard breakfast at 8am, ad libitum pizza meal at 13.00 hours as lunch.
Kudiganti et al. [74]	2016	RCT	Capsule	2 x 1	30 minutes before breakfast and dinner	Vegetarian or non-vegetarian diet of approximately 8368 kJ / 2000 kcal/day consisting of 17 % protein, 25 % fat and 58 % carbohydrate and 30 minutes walk for 5 days per week.
Kuriyan et al. [66]	2007	RCT	Capsule	2 x 1	n.r.	Subjects were provided with standard health advice on diet and physical activity targeted to achieve a weight loss of about 5 to 10 % body weight over the study period.
Lejeune et al. [59]	2003	RCT	Capsule	3 x 3	During breakfast, lunch and dinner	Very-low-energy diet (Modifast®; Novartis Nutrition, Breda, The Netherlands) during 4 weeks before the supplementation period:<br>3 sachets per day, dissolved in water to obtain a milk shake, pudding, soup or muesli. Vegetables and fruits were allowed in addition. The aim was a body-weight loss of at least 4 kg over 4 weeks.
Mangine et al. [55]	2012	RCT	Capsule	1 x 1 and 1 x 2	60 min before lunch (one capsule) and dinner (two capsules)	If the subject's daily caloric intake was at or above their total energy expenditure, they were recommended a diet that was 30 % or 2092 kJ / 500 kcal per day (whichever was greater) less than what they had been consuming. However, the maximum restriction did not exceed 41841 kJ / 1000 kcal per day.<br>Regular exercise schedule including at least 3 days per week, for 30 minutes per day at an intensity that that would elicit between 60 to 75% of the subject'smaximum heart rate.

Supplementary Materials

Author	Year	Type of study	Form	Daily intake	Time point of intake	Diet / exercise
Rondanelli et al. [64]	2013	RCT	Capsule	1 x 2	1 hour before lunch	For 3 days before the test day: Isoenergetic diet to maintain body weight by providing 55 % of energy as carbohydrates, 15 % as protein, and 30 % as fat. Test day: Arrival after 12 hours of fasting and abstinence from water, Consumption of a standard breakfast at 9.00 hours (3 slices of whole meal bread, a cup of skimmed milk [150 ml], 1 teaspoon of jam) and of a standard lunchtime mal at 12.00 hours (80 g rice, 80 g ham, 50 g white bread, 100 g lettuce, 150 g apple, 15 g olive oil and 500 ml tap water).
Rondanelli et al. [72]	2011	RCT	Tablets	3 x 1	Before breakfast, lunch, dinner	Restriction of their daily energy intake by a moderate amount, which is 3344 kJ/day (799 kcal) lower than the daily requirement based on World Health Organization criteria, with a regimen that maintained 25 % of energy from fats, 60 % of energy from carbohydrates and 15 % of energy from proteins. Furthermore, the subjects refrained from any form of exercise for 48 hours before the study.
Rondanelli et al. [56]	2009	RCT	Capsule	2 x 1	Before lunch and dinner	Restriction of their daily energy intake by a moderate amount, 3344 kJ/day (799 kcal) less than daily requirements based on World Health Organization criteria, with a regimen that maintained 25 % of energy from fat, 60 % of energy from carbohydrates and 15 % of energy from proteins.
Roshan et al. [63]	2018	RCT	Capsule	2 x 1	With the main meals	No modification of their physical activity and salt intake. Dietary plan with weight loss recommendations to amend the nutritional habits (30 % total fat, 18 % protein and 52 % carbohydrate).
Urbina et al. [58]	2017	RCT	Pill	1 x 1	After breakfast but before lunch (no empty stomach)	No alteration of diet, avoidance of foods containing chili pepper (i.e., serrano, cayenne, poblano, ancho, jalapeno, etc.) throughout the intervention. Test day: Arrival after 12 hours of fasting and 24 to 48 hours of no strenuous physical activity.
Westerterp-Plantenga et al. [54]	2005	RCT	Capsule	3 x 2	Before each meal	Very low energy diet intervention (2.1 MJ/day, Modifast®) during 4 weeks before the supplementation period: 3 sachets per day, dissolved in water to obtain a milk shake, pudding, soup or muesli. The diet was a protein-enriched formula diet, containing 50 g of carbohydrates, 52 g of protein, 7 g of fat and a micronutrient content that met the Dutch recommended daily allowance. Vegetables and fruit were allowed in addition to Modifast®. The aim was a body weight loss of at least 4 kg over 4 weeks.
Alkhatib et al. [65]	2015	Co-RCT	Capsule	1 x 4	Before 120 minutes resting	No intake of supplements for the duration of the study and no strenuous exercise or alcohol and caffeine consumption for at least 24 hours before each test. Test day: 30 minutes exercise cycling test at their individually determined Fatmax intensity.
Alkhatib et al. [28]	2017	Co-RCT	Capsule	1 x 3	150 minutes before exercise	No intake of supplements for the duration of the study and no strenuous exercise or alcohol and caffeine consumption for at least 24 hours before each test. Test day: 30 minutes exercise cycling test at their individually determined Fatmax intensity.
Fernandes et al. [51]	2018	Co-RCT	Capsule	1 x 2	With liquid test meal	72 hours before test day: no alcohol or beverages containing caffeine (coffee, cola and mate, black or green tea). The day before test day: Standardized dinner, delivered by researchers to avoid alteration in the production of appetite related hormones on the day of the experiment, no physical activity, maintenance of the usual diet. Test day: Arrival after 12 hours of subsequent fasting.
Greenberg et al. [60]	2012	Co-RCT	Beverage	1 x 1	Afternoon	During the study period, participants were asked: 1) To keep diet, exercise, and alcohol intake stable 2) Not to consume caffeinated drinks such as coffee, tea, sodas, or sports drinks; caffeinated medications; caffeinated diet or energy supplements; or decaffeinated coffee 3) Not to smoke

Supplementary Materials

Author	Year	Type of study	Form	Daily intake	Time point of intake	Diet / exercise
						4) Not to use alcohol<br>5) Not perform exercise during the 48 hours prior to each laboratory visit<br>6) To eat an identical small breakfast at the same time of day, with no snacks or lunch prior to each lab visit
Greenberg et al. [75]	2016	Co-RCT	Beverage	n.r.	n.r.	Between laboratory sessions, participants were asked:<br>1) To eat similar quantities of foods that contained constituents from the same food groups and with similar amounts of macronutrients during the same periods of the day after midnight of the day before each laboratory session<br>2) To avoid chocolate or cocoa beverages, tea, coffee, or other caffeinated drinks or tobacco or nicotine products for the duration of the study<br>3) To refrain from exercise and use of alcohol or psychotropic drugs during the 48 hours before laboratory sessions
Hao et al. [73]	2017	Co-RCT	Capsule	1 x 1	With breakfast	Before test day: consumption of the same dinner before each of the 3 test days.<br>Test day: arrival after an overnight fast, breakfast meal with 1150 kJ / 275 kcal; 50 % carbohydrate; 30 % fat and 20 % protein.
Hochkogler et al. [57]	2014	Co-RCT	Beverage	1 x 1	2 hours before breakfast	Standardized breakfast: four rolls, 3 slices of bread, 100 g strawberry jam, 60 g honey, 4 slices of ham, 4 slices of cheese, 180 g yogurt, 80 g creamery butter, 20 g sugar, 40 g coffee creamer, 200 ml water, and 200 ml coffee or tea (total energy content of 12.1 MJ / 2890 kcal; 355 g carbohydrates, 126 g fats, and 80 g proteins).<br>Test day: arrival after an overnight fast.
Hochkogler et al. [69]	2017	Co-RCT	Beverage	1 x 1	2 hours before breakfast	Standardized breakfast: 4 rolls, 3 slices of dark bread, 100 g strawberry jam, 60 g honey, 4 slices of ham, 4 slices of cheese, 180 g berry yogurt, 80 g creamery butter, 20 g sugar, 40 g coffee creamer, 200 ml of tea or coffee and water ad libitum (total energy content of 12.1 MJ / 2890 kcal; 335 g carbohydrates, 126 g fats, and 79.2 g proteins).<br>Test day: arrival after an overnight fast.
Janssens et al. [21]	2014	Co-RCT	Beverage	3 x 1	With breakfast, lunch and dinner	2 days prior to each session: standardized diet in order to be fed in energy balance (energy % protein/fat/carbohydrate: 15/30/55), and same macronutrient proportions as during the experiment, maintenance of habitual activity level and no consumption of alcohol.<br>The day before test day: No caffeine consumption after 22.00 hours.
Josic et al. [52]	2010	Co-RCT	Beverage	1 x 1	With breakfast	Test day: arrival after a 10 hours overnight fast.<br>Smoking, snuff taking and medication were prohibited in the morning prior to and during the test.
Mennella et al. [70]	2016	Co-RCT	Pudding	1 x 1	With breakfast	The day before test day: standardized dinner until 22.00 hours.<br>Test day: arrival after an overnight fast, consumption of the pudding (259 kJ / 62 kcal; 9.5 g carbohydrates, 5.5 g protein, 0.2 g fat) and a standardized breakfast (1314 kJ / 314 kcal; 45.1 g carbohydrates, 8.7 g protein, 10.6 g fat).
Panek-Shirley et al. [61]	2018	Co-RCT	Beverage	1 x 1	30 minutes before breakfast	1 day before test day: no beverages and foods containing caffeine, consumption of only plain water as a beverage and no vigorous exercise.<br>Test day: 30 minutes after the supplementation, participants were presented with an ample North American style buffet breakfast (ca. 33 MJ / 7878 kcal).
Reinbach et al. [53]	2009	Co-RCT	Beverage or capsule	3 x 1	Before each meal	Participants came fasting in the morning and were served standardized breakfast and lunch during weekdays, whereas they prepared their usual food in the evenings and weekends at home.

Supplementary Materials

Author	Year	Type of study	Form	Daily intake	Time point of intake	Diet / exercise
						Positive energy balance: 20 % of their individually calculated daily energy requirement for breakfast (2013 kJ / 481 kcal) and 40 % for lunch (4007 kJ / 958 kcal), additionally ad libitum dinner. Negative energy balance: 10 % of their individually calculated daily energy requirement for breakfast (1008 kJ / 241 kcal) and 15 % for lunch (1509 kJ / 361 kcal), additionally ad libitum dinner. Normally: 15 % of energy requirement for breakfast and 30 to 35 % for lunch.
Schubert et al. [62]	2014	Co-RCT	Capsule	2 x 1	With breakfast and at mid-morning	1 day before test day: standardization of food intake (dinner with 3073 ± 506 kJ / 734 kcal ± 121 kcal , 109 ± 20 g carbohydrates, 25 ± 4 g protein and 21 ± 4 g fat, equivalent to ~ 30 % of daily energy needs). No strenuous exercise, no alcohol, no foods naturally enriched in 13C (corn and corn-based products, kiwi, pineapple, cane sugar) 2 days before test day: no consumption of anything containing caffeine or known to influence caffeine metabolism (i.e. cruciferous vegetables, charcoal-broiled beef, aspirin, and cimetidine). Test day: arrival after an overnight-fast (nothing than water after 22.00 hours).
Shin et al. [76]	2015	Co-RCT	Capsule	I1: 1 x 1 and 1 x 2 placebo I2: 1 x 3	With breakfast	Test day: arrival after an overnight fast, standardized breakfast: 2 MJ high starch, low-polyphenol (185 g white bread containing 83 g polysaccharide starch, 1943 kJ / 464 kcal). Washout period: free to resume usual diet and exercise patterns.

[1] Co-RCT, crossover randomized controlled trial; I, intervention; n.r., not reported; RCT, randomized controlled trial.

Supplementary Materials

Table S6: Primary outcomes [1].

Study.	Year	Type of study	Plant extract(s) of [2]	Appetite	Hunger	Satiety	Fullness
Auvichayapat et al. [48]	2008	RCT	*Camellia sinensis* (green tea)	n.r.	n.r.	0	n.r.
Boix-Castejon et al. [71]	2018	RCT	*Hibiscus sabdariffa* + *Aloysia citriodora*	n.r.	+	+	+
Diepvens et al. [49]	2005	RCT	*Camellia sinensis* (green tea)	0	-	0	0
Dostal et al. [50]	2017	RCT	*Camellia sinensis* (green tea)	n.r.	0	0	0
Gonzalez et al. [68]	2018	RCT	*Crocus sativus* + *Citrus paradisi*	n.r.	0	0	0
Gout et al. [67]	2010	RCT	*Crocus sativus*	n.r.	+	0	n.r.
Kazemipoor et al. [15]	2016	RCT	*Carum carvi*	+	n.r.	n.r.	n.r.
Kudiganti et al. [74]	2016	RCT	*Sphaeranthus indicus* + *Garcinia mangostana*	n.r.	n.r.	+	n.r.
Kuriyan et al. [66]	2007	RCT	*Caralluma adscendens var. fimbriata*	n.r.	+	n.r.	0
Lejeune et al. [59]	2003	RCT	*Capsicum annuum*	n.r.	0	0	n.r.
Mangine et al. [55]	2012	RCT	*Camellia sinensis* (green tea)	n.r.	0	0	n.r.
Rondanelli et al. [64]	2013	RCT	*Camellia sinensis* (green tea) + *Capsicum annuum* + *Piper nigrum* + *Fucus vesiculosus* + *Allium sativa*	n.r.	n.r.	+	+
Rondanelli et al. [72]	2011	RCT	*Phaseolus vulgaris* + *Cynara scolymus*	n.r.	n.r.	++	n.r.
Rondanelli et al. [56]	2009	RCT	*Camellia sinensis* (green tea)	+	n.r.	n.r.	n.r.
Roshan et al. [63]	2018	RCT	*Coffea* sp.	+	n.r.	n.r.	n.r.
Urbina et al. [58]	2017	RCT	*Capsicum annuum*	0	n.r.	n.r.	n.r.
Westerterp-Plantenga et al. [54]	2005	RCT	*Camellia sinensis* (green tea)	n.r.	0	0	n.r.
Alkhatib et al. [65]	2015	Co-RCT	*Camellia sinensis* (green tea) + *Ilex paraguariensis* (Yerba Maté) + *Paullinia cupana* + *Coffea* sp. + *Serenoa repens* + *Polygonum multiflorum* + *Eleutherococcus senticosus* + *Capsicum annuum* + *Pausinystalia yohimbe*	n.r.	n.r.	0	n.r.
Alkhatib et al. [28]	2017	Co-RCT	*Ilex paraguariensis* (Yerba Maté)	n.r.	0	n.r.	0
Fernandes et al. [51]	2018	Co-RCT	*Camellia sinensis* (green tea)	n.r.	0	n.r.	+
Greenberg et al. [75]	2016	Co-RCT	I1: *Theobroma cacao* (nonalkalized cocoa mixture)	n.r.	0	0	+
			I2: *Theobroma cacao* (epicatechin)	n.r.	+	+	+
			I3: *Theobroma cacao* (procyanidins)	n.r.	n.r.	n.r.	n.r.
Greenberg et al. [60]	2012	Co-RCT	I1: *Coffea* sp. (caffeine)	n.r.	0	0	n.r.
			I2: *Coffea* sp. (coffee caffeinated)	n.r.	0	0	n.r.
			I3: *Coffea* sp. (coffee decaffeinated)	n.r.	++	0	n.r.
Hao et al. [73]	2017	Co-RCT	*Salacia chinensis*	n.r.	0	0	0
Hochkogler et al. [57]	2014	Co-RCT	*Capsicum annuum*	n.r.	+	n.r.	n.r.
Hochkogler et al. [69]	2017	Co-RCT	*Eriodictyon californicum*	n.r.	0	n.r.	n.r.
Janssens et al. [21]	2014	Co-RCT	*Capsicum frutescens* + *Capsicum annuum*	n.r.	0	+	+

Supplementary Materials

Study.	Year	Type of study	Plant extract(s) of [2]	Appetite	Hunger	Satiety	Fullness
Josic et al. [52]	2010	Co-RCT	*Camellia sinensis* (green tea)	n.r.	0	+	+
Mennella et al. [70]	2016	Co-RCT	*Gentiana lutea*	n.r.	0	0	0
Panek-Shirley et al. [61]	2018	Co-RCT	*Coffea* sp.	n.r.	0	n.r.	0
Reinbach et al. [53]	2009	Co-RCT	I1: *Capsicum annuum* (cayenne)	n.r.	+	+	+
			I2: *Camellia sinensis* (green tea)	n.r.	+	0	+
			I3: *Capsicum annuum* (CH-19 sweet pepper)	n.r.	0	0	0
			I4: *Capsicum annuum* (cayenne) + *Camellia sinensis* (green tea	n.r.	++	++	++
Schubert et al. [62] [3]	2014	Co-RCT	I1: *Coffea* sp. (caffeine)	n.r.	0	n.r.	0
			I2: *Coffea* sp. (caffeine + decaffeinated coffee)	n.r.	0	n.r.	0
Shin et al. [76]	2015	Co-RCT	*Vitis vinifera*	n.r.	0	n.r.	0

[1] Co-RCT, crossover randomized controlled trial; n.r., not reported; RCT, randomized controlled trial. [2] For accepted scientific name and exact composition see Table S4. [3] Intervention group with psyllium husk and decaffeinated coffee excluded due to our exclusion criteria (seeds). "-" – Significant treatment effect ($p < 0.05$) in favour of the placebo group. "0" – No significant treatment effect ($p > 0.05$). "+" – Some evidence: significant treatment effect ($p < 0.05$) in favour of the intervention group in at least one follow-up comparison. "++" – Strong evidence: significant treatment effect ($p < 0.05$) in favour of the intervention group in all follow-up comparisons.

Supplementary Materials

Table S7: SIGN checklist for randomized controlled trials [46] [1].

Author	Year	Type of study	1.1	1.2	1.3	1.4	1.5	1.6	1.7	1.8 I/C, %	1.9	1.10	2.1
Auvichayapat et al. [48]	2008	RCT	Y	CS	N	CS	Y	Y	Y	0/0	Y	NA	0
Boix-Castejon et al. [71]	2018	RCT	Y	Y	Y	Y	Y	Y	Y	10/15	Y	NA	++
Diepvens et al. [49]	2005	RCT	Y	CS	N	Y	Y	Y	Y	0/0	Y	NA	+
Dostal et al. [50]	2017	RCT	Y	Y	N	Y	Y	Y	Y	12/0	N	NA	+
Gonzalez et al. [68]	2018	RCT	Y	CS	N	Y	CS	Y	Y	0/0	Y	NA	0
Gout et al. [67]	2010	RCT	Y	Y	Y	Y	Y	Y	Y	0/3.3	N	NA	++
Kazemipoor et al. [15]	2016	RCT	Y	CS	N	Y	Y	Y	Y	11/17	N	NA	0
Kudiganti et al. [74]	2016	RCT	Y	Y	Y	Y	Y	Y	Y	3/7	N	NA	++
Kuriyan et al. [66]	2007	RCT	Y	CS	N	Y	Y	Y	Y	19/19	N	NA	0
Lejeune et al. [59]	2003	RCT	Y	CS	N	Y	Y	Y	Y	n.r.	N	NA	0
Mangine et al. [55]	2012	RCT	Y	CS	N	Y	Y	Y	Y	36/36	N	NA	0
Rondanelli et al. [64]	2013	RCT	Y	Y	N	Y	Y	Y	Y	0/0	Y	NA	++
Rondanelli et al. [72]	2011	RCT	Y	Y	Y	Y	Y	Y	Y	0/5	N	NA	++
Rondanelli et al. [56]	2009	RCT	Y	Y	N	Y	Y	Y	Y	6/27	Y	NA	++
Roshan et al. [63]	2018	RCT	Y	Y	Y	Y	Y	Y	Y	16/12	N	NA	++
Urbina et al. [58]	2017	RCT	Y	CS	N	Y	Y	Y	Y	23/44/24	N	NA	0
Westerterp-Plantenga et al. [54]	2005	RCT	Y	CS	N	Y	Y	Y	Y	0/0	Y	NA	+
Alkhatib et al. [65]	2015	Co-RCT	Y	CS	N	Y	CS	Y	Y	0	Y	NA	+
Alkhatib et al. [28]	2017	Co-RCT	Y	CS	N	Y	CS	Y	Y	42.9	N	NA	0
Fernandes et al. [51]	2018	Co-RCT	Y	Y	N	Y	CS	Y	Y	8/0	N	NA	+
Greenberg et al. [75]	2016	Co-RCT	Y	Y	N	Y	CS	Y	Y	6.7	N	NA	+
Greenberg et al. [60]	2012	Co-RCT	Y	CS	N	Y	CS	Y	Y	0	Y	NA	+
Hao et al. [73]	2017	Co-RCT	Y	Y	N	Y	CS	Y	Y	11.1	Y	NA	++
Hochkogler et al. [57]	2014	Co-RCT	Y	CS	CS	CS	CS	Y	Y	0	Y	NA	0
Hochkogler et al. [69]	2017	Co-RCT	Y	CS	N	Y	CS	Y	Y	29.2	N	NA	0
Janssens et al. [21]	2014	Co-RCT	Y	CS	N	Y	CS	Y	Y	21.1	N	NA	0
Josic et al. [52]	2010	Co-RCT	Y	CS	N	N	CS	Y	Y	6.7	N	NA	0
Mennella et al. [70]	2016	Co-RCT	Y	CS	N	Y	CS	Y	Y	CS	CS	NA	0
Panek-Shirley et al. [61]	2018	Co-RCT	Y	Y	N	Y	CS	Y	Y	5.7	N	NA	+
Reinbach et al. [53]	2009	Co-RCT	Y	Y	N	Y	CS	Y	Y	CS	CS	NA	0
Schubert et al. [62]	2014	Co-RCT	Y	Y	Y	Y	CS	Y	Y	33.3	N	NA	++
Shin et al. [76]	2015	Co-RCT	Y	CS	Y	Y	CS	Y	Y	0	Y	NA	++

[1] C, control; Co-RCT, crossover randomized controlled trial; CS, can't say; I, intervention; N. no; NA, not applicable; RCT, randomized controlled trial; Y, yes. 1.1 – appropriate and clearly focused question; 1.2 – assignment is randomized; 1.3 - adequate concealment method; 1.4 – blinding; 1.5 – groups are similar at baseline; 1.6 – treatment under investigation is the only difference; 1.7 – all relevant outcomes are measured in a standard, valid and reliable way; 1.8 - percentage of drop-outs in each treatment arm, 1.9 – intention to treat analysis; 1.10 – various sites are comparable; 2.1 – risk of bias: high quality (++): most of the criteria have been fulfilled (if at most one criterion was answered with a "no" or "can't say"); acceptable quality (+): some criteria fulfilled (if at most two criteria were answered with a "no" or "can't say"); low quality (0): few criteria fulfilled (if at most four criteria were answered with a "no" or "can't say"); unacceptable (-): study rejected (if more than four criteria were answered with a "no" or "can't say"). For Co-RCTs criterion 1.5 was not applicable and therefore not considered for the final rating.
